# Tuberculosis in Monkey

**Published:** 1891-06

**Authors:** 


### TUBERCULOSIS IN MONKEY.

### *Rupture of Uterus.*

About the first of April a female rhesus monkey (*Macacus rhesus*) was noticed to have a cough. Simple remedies were given it, and on the tenth she gave birth to a fœtus about five months old, which was sent to Prof. B. G. Wilder, of Ithaca. After that time the monkey continued to droop, and died on the 2nd of May. A post mortem by Dr. Geo. S. Huntington revealed the following: Right lung, excessive tuberculosis, involving large portions of all lobes; bronchial (right) and posterior mediastinal glands tubercular; tubercular abscess in bronchial gland opening into bronchus just beyond bifurcation of trachea; left lung and other thoracic contents healthy; abdominal viscera healthy; uterus somewhat enlarged and in an advanced condition of involution; tissue exceedingly friable. There is a rupture on posterior aspect of fundus transverse, and a second tear in anterior uterine wall at about the middle. The edges of these ruptures were covered with a small amount of coagulated blood. No blood or fluid in peritoneal cavity. No peritonitis. If these tears in the uterus occurred ante mortem they must have taken place immediately before death in order to account for the absence of blood in peritoneal cavity, and the absence of inflammatory effusion. It is possible that this occurred in some way, post mortem, in handling the body before opening it, in the exceedingly friable condition of uterus. The condition of right lung would be a sufficent cause of death. It is possible that the uterine rupture occurred during life and produced death by shock in an an enfeebled animal.*

* Such cases occur in bitches. *Ed.*

